# New considerations on pathways involved in acute traumatic coagulopathy: the thrombin generation paradox

**DOI:** 10.1186/s13017-019-0276-8

**Published:** 2019-12-12

**Authors:** Cedric Gangloff, Fanny Mingant, Michael Theron, Hubert Galinat, Ollivier Grimault, Yves Ozier, Karine Pichavant-Rafini

**Affiliations:** 1 ORPHY Laboratory EA4324, Université de Bretagne Occidentale, Brest, France; 2 Department of Emergency Medicine, CHU Pontchaillou, Rennes, France; 3 Department of Biology and Hemostasis, CHRU Cavale Blanche, Brest, France; 4 Department of Emergency Medicine, Hôpital de la Cavale Blanche, Brest, France; 5 Department of Anaesthesiology, Hôpital de la Cavale Blanche, Brest, France

**Keywords:** Trauma, Coagulation, Shock, Thrombin, Platelet, Model

## Abstract

**Abstract:**

**Background:**

An acute traumatic coagulopathy (ATC) is observed in about one third of severely traumatized patients. This early, specific, and endogenous disorder is triggered by the association of trauma and hemorrhage. The early phase of this condition is characterized by the expression of a bleeding phenotype leading to hemorrhagic shock and the late phase by a prothrombotic profile leading to multiple organ failure. The physiopathology of this phenomenon is still poorly understood. Hypotheses of disseminated intravascular coagulation, activated protein C-mediated fibrinolysis, fibrinogen consumption, and platelet functional impairment were developed by previous authors and continue to be debated. The objective of this study was to observe general hemostasis disorders in case of ATC to confront these hypotheses.

**Method:**

Four groups of 15 rats were compared: C, control; T, trauma; H, hemorrhage; and TH, trauma and hemorrhage. Blood samples were drawn at baseline and 90 min. Thrombin generation tests, platelet aggregometry, and standard hemostasis tests were performed.

**Results:**

Significant differences were observed between the baseline and TH groups for aPTT (17.9 ± 0.8 s vs 24.3 ± 1.4 s, *p* < 0.001, mean ± SEM), MAP (79.7 ± 1.3 mmHg vs 43.8 ± 1.3 mmHg, *p* < 0.001, mean ± SEM), and hemoglobin (16.5 ± 0.1 g/dL vs 14.1 ± 0.3 g/dL, *p* < 0.001, mean ± SEM), indicating the presence of an hemorrhagic shock due to ATC. Compared to all other groups, coagulation factor activities were decreased in the TH group, but endogenous thrombin potential was (paradoxically) higher than in group C (312 ± 17 nM/min vs. 228 ± 23 nM/min; *p* = 0.016; mean ± SEM). We also observed a subtle decrease in platelet count and function in case of ATC and retrieved an inversed linear relationship between fibrinogen concentration and aPTT (intercept, 26.53 ± 3.16; coefficient, − 3.40 ± 1.26; adjusted *R*^2^: 0.1878; *p* = 0.0123).

**Conclusions:**

The clinical-biological profile that we observed, combining normal thrombin generation, fibrinogen depletion, and a hemorrhagic phenotype, reinforced the hypothesis of activated protein C mediated-fibrinolysis. The key role of fibrinogen, but not of the platelets, was confirmed in this study. The paradoxical preservation of thrombin generation suggests a protective mechanism mediated by rhabdomyolysis in case of major trauma. Based on these results, we propose a new conception concerning the pathophysiology of ATC.

## Background

One third of severe trauma patients present an acute traumatic coagulopathy (ATC) upon hospital admission. ATC is an acute, specific, and endogenous phenomenon triggered by the association of trauma and hemorrhage. Its presence is associated with higher mortality and transfusion rates [1, 2]. External factors, such as hypothermia, anti-thrombotic, and dilution, can reinforce this coagulopathy [3]. In the first hours, hemorrhage and macrocirculatory impairment are responsible for an early mortality peak. In the following days, delayed mortality due to microcirculatory defects—reflecting a prothrombotic state—is observed [4]. The physiopathology of ATC is still debated due to its complexity, but the involvement of an imbalance between pro- and anti-coagulant pathways, platelets, and endothelium are currently agreed upon [5–8]. However, interactions between them remain unclear. Some authors hypothesized an increased production of activated protein C (aPC) to explain the emergence of hyper-fibrinolysis in case of ATC, but this hypothesis is contradicted by studies reporting normal or increased thrombin generation after severe trauma while it should be reduced due to the inhibitory effect of aPC on FVa and FVIIIa [9–11]. This argument has been echoed by advocates of an another hypothesis: ATC would be a disseminated intravascular coagulation (DIC) associated with an early fibrinolytic phenotype explained by an endothelial release of tissue plaminogen activator (t-PA) [12–14]. These authors argued that aPC concentrations observed in case of ATC were insufficient to repress FVa and FVIIIa and lengthen prothrombin time (PT) in vitro [15, 16]. Contradictory studies reported the absence of clinical criteria of DIC [9] or decreased thrombin generation after trauma [17]. Observation of thrombin generation on a reliable animal model of ATC, not influenced by confounding factors, would therefore make possible to confront these two hypotheses. Another unresolved concern is the role of platelet on ATC. Indeed, Jacoby et al. observed a rise in platelet-activation markers associated with hypo-functional platelets, suggesting the presence of a refractory state due to trauma [18, 19]. In addition, a loss of platelet function was identified as a predictive factor of mortality in this context [20, 21]. These concerns explain why thrombin generation test (TGT) and platelet aggregometry were identified as potentially useful to explore ATC [22–24]. The objective of this study was to explore the general hemostasis disorders involved in ATC’s genesis to confront them with hypotheses proposed to explain its pathophysiology.

## Methods

### Animals

Sixty adult Sprague-Dawley rats (430–650 g, Janvier SAS, Le Genest St. Isla, France) were housed in a controlled environment (temperature 21 ± 1 °C, relative humidity 27 ± 16%, 12–12 h light-dark cycle). All procedures were conducted following a protocol approved by the French ministry of agriculture (APAFIS#5194-2016042513131045) and the local university animal research ethics committee. Procedures were in line with the guide for the care and use of laboratory animals published by the US National Institute of Health [25].

### Preparation

Animals were anesthetized with an intraperitoneal injection of ketamine (100 mg/Kg, Virbac, Carros, France) and xylazine (10 mg/kg, Virbac, Carros, France). They were then placed on a warming pad (Z31SY, Ascon technologic, Italy) to maintain central body temperature in a normal range (37.5 ± 0.5 °C). A 2-cm cervical incision was performed, followed by a tracheostomy (2-mm-diameter polyethylene tube). An arterial catheter (Leader Flex 22G, 0.7 × 40 mm, Vygon, France) was inserted in the right carotid. A venous catheter was inserted in the left jugular vein (Leader Flex 22G, 0.7 × 40 mm, Vygon, France) followed by a continuous intravenous infusion of ketamine (1 mg/kg/h, Virbac Inc., Carros, France).

### Experimental procedure

**Fig. 1:**
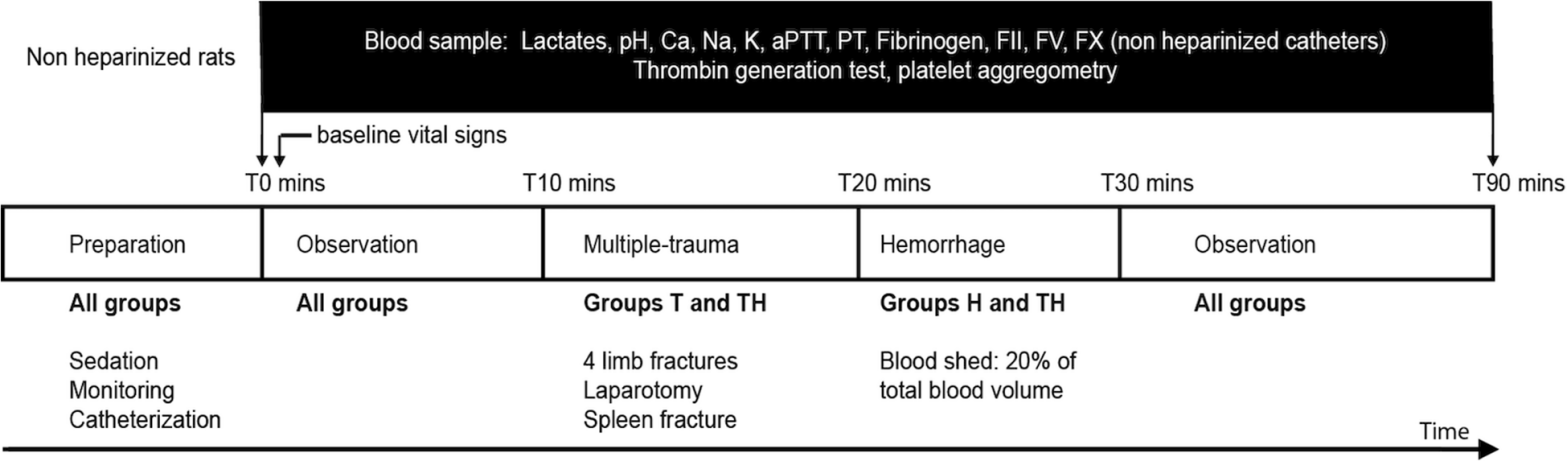
Experimental protocol. Group C, without trauma without hemorrhage; T, trauma without hemorrhage; H, hemorrhage without trauma; TH, hemorrhage with trauma (*n* = 15 in each group)

The experimental procedure is summarized in Fig. 1. Rats were allocated randomly to one of the four experimental groups (*n* = 15 per group): control (C), in which neither trauma nor hemorrhage was performed; trauma (T), in which trauma was performed but not hemorrhage; hemorrhage (H), in which hemorrhage was performed but not trauma; trauma and hemorrhage (TH), in which trauma and hemorrhage were performed. In groups H and TH, 20% of total blood mass was gently collected. In groups T and TH, multiple traumas were performed as follows: four closed limb fractures on the mid-height of the bone (two femurs, two humeri) at 90 angular degrees with pliers. A 4-cm median laparotomy, as well as four spleen crushings of 1 cm on the inferior border of the spleen, was done with surgical scissors and a needle holder.

### Blood samples

All blood samples were collected through the arterial catheter. Three 15-min centrifugations were performed to obtain poor platelet plasma: one at 1000 g and two at 3000 g (centrifuge 2–16 K, Sigma, Germany). Plasma was frozen at – 80 °C until measurements.

### Blood analysis

Arterial blood pH, lactate, and potassium concentrations were measured with a point-of-care analyzer (ABL80 FLEX, Radiometer, Copenhagen, Denmark). FII, FV, FX, fibrinogen, PT, and aPTT assays were performed on an automated analyzer (STA-R Evolution, Stago, Asnieres sur Seine, France). PT, aPTT times, and fibrinogen concentrations were measured with neoplastin Cl + 10, triniclot aPTTb, and STA liquid fib, respectively. Specific factor-depleted plasmas (Stago, Asnieres sur Seine, France) were used to determine coagulation factor activities. The thrombin generation test was performed using the Thrombinoscope CAT (Calibrated Automated Thrombogram, Maastricht, The Netherlands) assay according to the manufacturer’s instructions (Diagnostica Stago, Asnières, France) [7, 8]. Twenty microliters of plasma was incubated with 20 μL PPP-ReagentTM (containing 5 pM recombinant tissue factor and 4 μM phospholipids) for 10 min in round-bottomed 96-well black microplates. For each sample, a calibrator (Thrombin CalibratorTM) was run in parallel in order to correct the fluorescence signal for substrate consumption and plasma color variability. Thrombin generation was initiated by the addition of 20 μL of FluCa-KitTM). Fluorescence was detected by a Fluoroskan Ascent1 fluorimeter (Thermo Fischer Scientific, Waltham, MA), and the thrombin generation curves were analyzed by the thrombinoscope software (Thrombino- scope BV, Maastricht, The Netherlands). Thrombin generation curves was characterized by 5 parameters: “endogenous thrombin potential” (ETP), the area under the curve expressed in nM/min; “lagtime,” the length of time required before thrombin generation starts; “peak,” the highest thrombin concentration; “time to peak,” the length of time until peak; and “start tail,” the duration to end-point of thrombin generation. Platelet aggregometry was performed with a Multiplate analyzer (Verum Diagnostica GmbH, Munich, Germany) in a whole blood sample, as described by the manufacturer. Three platelet agonists specific to three pathways were tested: “PAR-4 test” (70 mmol/L, PAR-4 receptor, SIGMA, St. Louis, USA); “ADP test” (10 mmol/L, ADP receptor, Roche Diagnostics GmbH, Sandhofer Mannheim, Germany); and “COLLtest” (1.4 μg/ml, collagen receptor, Roche Diagnostics GmbH, Sandhofer Mannheim, Germany). The value recorded was the area under the curve (AUC).

### Statistical analysis and graphics

Statistical analyses were performed with “SPSS statistics for Macintosh” software version 21 (I.B.M. corp., Armonk, NY, 2012). Line graphs, boxplots, and histograms were generated using “Prism 7 for Mac OS X” version 7.0a (GraphPad Software, La Jolla, USA, 2016). At the time of the first sampling, the results were pooled and compared with the sampling performed at 90 min for each group (C, T, H, and TH). One-way ANOVA with adequate post hoc tests was used to compare means between groups. Results were expressed as the mean ± standard error of the mean (SEM). A *p* value < 0.05 was considered statistically significant.

## Results

### Markers of ATC

**Fig. 2:**
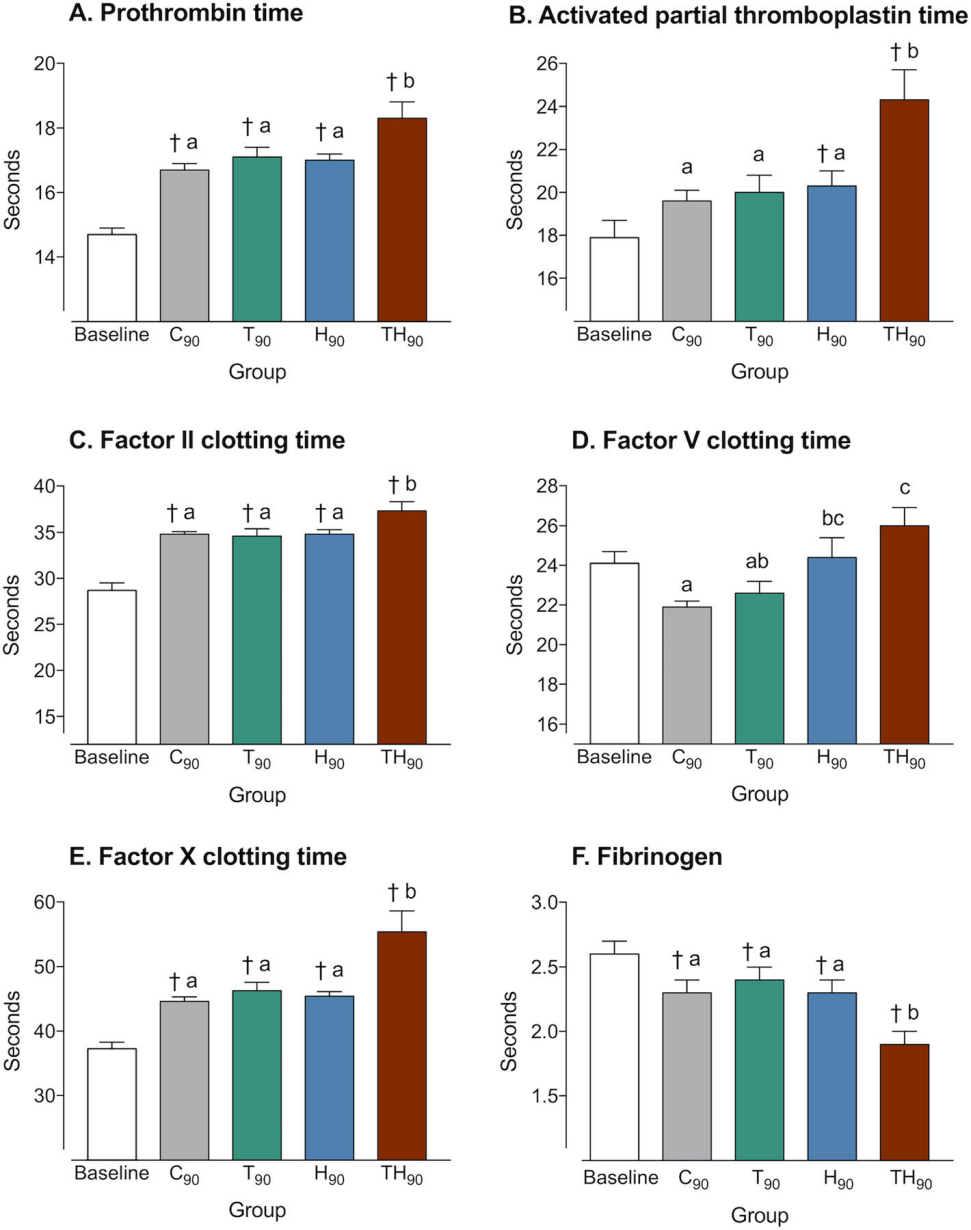
Markers of ATC and coagulation factors. Baseline, pooled values from all groups at 0 min; C_90_, control at 90 min; T_90_, trauma at 90 min; H_90_, hemorrhage at 90 min; TH_90_, trauma and hemorrhage at 90 min. Data are presented as mean ± SEM. *ANOVA I significance was designated at the *p* < 0.05 level of confidence. ^†^Significantly different from baseline. Letter differences indicate statistical differences between groups at 90 min

The mean PT was statistically higher than baseline at the end of the experimentation in groups C, T, H, and TH. At 90 min, the group TH had a PT significantly longer than the C, T, and H groups (Fig. 2a). Similar trends were observed for aPTT (Fig. 2b).

### Trauma, shock, and hemorrhage markers

Significant differences were observed between the baseline and TH group for MAP, base excess, potassium, and hemoglobin (Table 1).

**Table 1:** Biological assays at baseline and after procedure in each group

Biological test	Baseline	Group C_90_	Group T_90_	Group H_90_	Group TH_90_	*p* value
Trauma, shock, and hemorrhage						
pH	7.39 ± 0.01	7.36 ± 0.04 ^a^	7.41 ± 0.01 ^ab^	7.43 ± 0.02 ^b^	7.45 ± 0.05 ^b†^	0.049*
Base excess (mmol/L)	0.4 ± 0.2	− 2.4 ± 0.3 ^a†^	− 5.4 ± 0.8 ^b†^	− 2.6 ± 0.5 ^a†^	− 8.03 ± 0.9 ^c†^	< 0.001*
PCO_2_	43 ± 0.86	34.9 ± 2	29.6 ± 1.8 ^†^	30.6 ± 1.9 ^†^	30.8 ± 7.9 ^†^	< 0.001*
Bicarbonates	24.7 ± 0.8	19.1 ± 0.9 ^a†^	18.1 ± 0.8 ^ab†^	20.4 ± 0.5 ^a†^	15.5 ± 0.8 ^†b^	< 0.001*
Lactates (mmol/L)	0.5 ± 0.1	1 ± 0.1 ^a†^	0.9 ± 0.2 ^a†^	1.3 ± 0.2 ^a†^	2 ± 0.4 ^b†^	< 0.001*
Potassium (mmol/L)	3.51 ± 0.05	4.55 ± 0.22 ^a†^	4.68 ± 0.13 ^a†^	4.39 ± 0.13 ^a†^	5.95 ± 0.33 ^b†^	< 0.001*
Hemoglobin (g/dL)	16.5 ± 0.1	14.8 ± 0.2 ^ab†^	15.6 ± 0.3 ^a†^	13.4 ± 0.6 ^c†^	14.1 ± 0.3 ^bc†^	< 0.001*
Thrombin generation						
Endogenous thrombin potential (nM/min)	269 ± 9	228 ± 23 ^a^	262 ± 15 ^ab^	297 ± 17 ^ab^	312 ± 17 ^b^	0.016*
Lagtime(min)	2.07 ± 0.07	2.49 ± 0.10 ^†^	2.38 ± 0.08 ^†^	2.41 ± 0.14 ^†^	2.47 ± 0.09 ^†^	< 0.001*
Peak (nmol/L)	93.0 ± 5.2	60.5 ± 6.7^a†^	73.6 ± 5.7 ^ab†^	86.9 ± 6.8 ^b^	79.2 ± 6.6 ^ab^	0.009*
Time to peak (min)	4.0 ± 0.1	4.3 ± 0.1 ^a†^	4.1 ± 0.1 ^a^	4.1 ± 0.1 ^a^	4.5 ± 0.1 ^a†^	0.02*
Start tail(min)	53.6 ± 3.9	34.3 ± 4.2 ^a†^	42.2 ± 3.2 ^a^	52.8 ± 4.1 ^b^	40.9 ± 4.3 ^a^	0.02*
Platelets						
Platelets (platelets/L)	570 ± 8	517 ± 12 ^a†^	533 ± 26 ^a^	493 ± 17 ^a†^	525 ± 15 ^a†^	< 0.001*
PAR-4 (AUC)	74.7 ± 2.5	69.4 ± 4.1	83.5 ± 7.0	63.3 ± 5.7	70.3 ± 4.7	0.146
ADP (AUC)	91.0 ± 1.8	90.9 ± 4.1	100.0 ± 5.7	87.3 ± 4.4	85.3 ± 4.3	0.214
COLL (AUC)	86.0 ± 2.0	77.4 ± 2.5 ^a†^	84.3 ± 2.8 ^a^	80.5 ± 3.6 ^a^	75.5 ± 4.0 ^a†^	0.047*

Data are presented as mean ± SEM. Baseline, pooled values from all groups at 0 min; C_90_, control at 90 min; T_90_, trauma at 90 min; H_90_, hemorrhage at 90 min; TH_90_, trauma and hemorrhage at 90 min. *ANOVA I significance was designated at the *p* < 0.05 level of confidence. ^†^Significant difference with baseline. Letter differences indicate statistical differences between groups. *N* = 15 per group

### Thrombin generation tests

At 90 min, the TH group exhibited statistically higher ETP when compared to group C (Table 1). At the end of the experimentation, lagtime, peak, time to peak, and start tail were statistically lower than baseline in group C. Trauma by itself induced no modification when compared with group C. Hemorrhage alone induced an increase in lagtime, peak amplitude, and start tail.

### Specific coagulation assays

In the control group, FII and FX times increased and fibrinogen concentration decreased when compared to baseline. In group TH, all of the measured parameters were modified: FII, FV, and FX times were longer than in group C, and the fibrinogen concentration was significantly lowered (Fig. 2c–f). A statistically significant inversed relationship between fibrinogen concentration and aPTT was observed (intercept, 26.53 ± 3.16; coefficient, − 3.40 ± 1.26; adjusted *R*^2^: 0.1878; *p* = 0.0123).

### Platelet count and aggregometry

Platelets slightly decreased from baseline in all groups at 90 min (Table 1). There was no effect of the different procedures at the end of the experimentation. Concerning aggregometry parameters, there was no statistical difference between groups C, T, H, or TH.

## Discussion

### Model relevance: this model reproduced the early phase of ATC

**Fig. 3:**
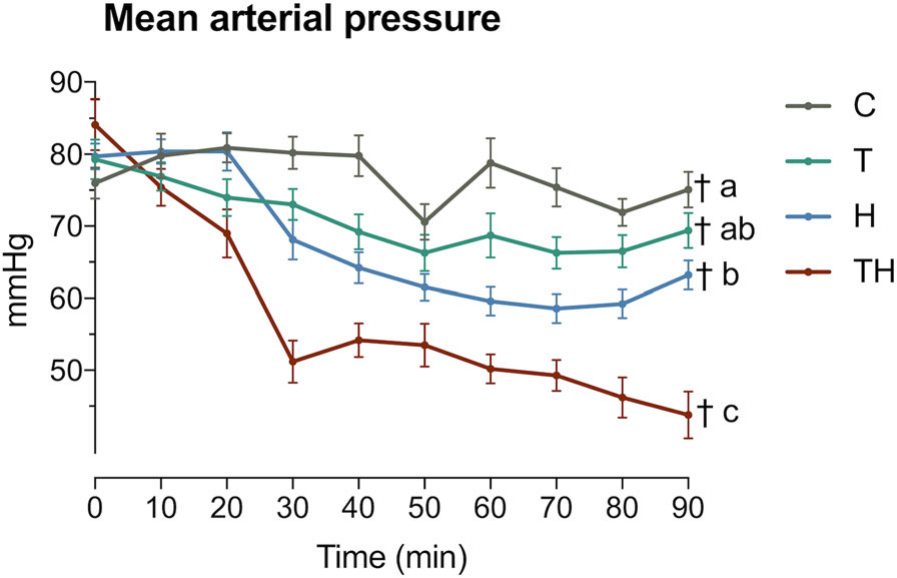
Evolution of MAP during the time in each group. C, control; T, trauma; H, hemorrhage; TH, trauma and hemorrhage, *n* = 15 per group. Values represent mean ± SEM. ^†^Significantly different from baseline. Letter differences indicate statistical differences between groups at 90 min

The mean PT and aPTT were statistically longer in group TH than in all other groups at the end of the experimentation. These coagulation disorders resulted in a bleeding phenotype because MAP remained lower in this group at 90 min (Fig. 3). Persisting hypotension was associated with an increase in lactate, reflecting an energetic imbalance in this context (Table 1). This state is called “uncompensated shock” [26]. Shock leaded to metabolic acidosis, as measured by the decrease in base excess (Table 1). Bicarbonate buffer and alveolar hyperventilation were activated in this group. Indeed, bicarbonates and pCO_2_ decreased drastically, leading to a subtle rise in pH despite the presence of metabolic acidosis. In synthesis, the TH group reproduced the early phase of shock, without acidemia. Concerning potential bias, we did not use a fluid replacement that could dilute coagulation factors or antithrombotic injection to prevent clot formation, and we prevented hypothermia that could reduce clotting factor enzymatic protease activity. In consequences, the coagulation disorder observed in group TH fits all the characteristics defining ATC: an acute and endogenous coagulopathy specifically triggered by trauma and hemorrhage [18, 19].

### Early activation of pro- and anticoagulant pathways in case of ATC

#### The thrombin generation paradox: a statement

ETP reflects the total amount of thrombin that a plasma sample can generate under the action of pro- and anticoagulant drivers [27–30]. In the case of coagulation factor depletion, thrombin generation tends to decrease [31]. This coagulation profile was observed in group C. Indeed, FV, FX, and ETP decreased in this group, probably due to cervical incision, tracheostomy, and catheter insertions. Paradoxically, we observed higher ETPs in group TH than in group C at the end of the experimentation despite higher FV and FX times.

This clinical-biological profile combining a hemorrhagic phenotype, a depletion of coagulation factors, and the paradoxical preservation of thrombin generation must be confronted with the two hypotheses that have been in opposition for several years concerning ATC’s pathophysiology: trauma-related DIC and aPC-mediated fibrinolysis.

In the case of DIC, a major decrease in thrombin generation [32] and platelet count [12, 14] should be observed. In this experimentation, thrombin generation was preserved, and platelet count was only slightly decreased in group TH. These results are inconsistent with the DIC hypothesis. This assumption is reinforced by the observation of a marked decrease in fibrinogen concentration, as usually observed in case of aPC-mediated fibrinolysis, and supported by several studies reporting an increase in aPC in the presence of ATC [15, 16, 33]. But the inhibition of FVa and FVIIIa by aPC should lead to a decrease in thrombin generation, suggesting the existence of a protective mechanism.

In synthesis, we observed the preservation of normal thrombin generation despite a clinical-biological profile indicating aPC-mediated fibrinolysis. These results suggest the existence of a mechanism protecting thrombin generation against aPC. We called this phenomenon the “thrombin generation paradox.”

#### Pathways involved in the thrombin generation paradox: a hypothesis

We identified a mechanism that could explain this paradox. Indeed, the activity of the prothrombinase complex, which plays a crucial role in thrombin generation, is enhanced by two proteins whose plasma concentration increases in case of trauma-related hemorrhage: myosin and tissue factor (TF).

Major trauma leads directly to cellular damages by mechanical action on tissues [34]. Plasmatic rises in potassium, TF [35], and myosin [36, 37] due to cellular leakage are observed in this condition. This mechanism, called rhabdomyolysis or “crush syndrome,” is potentialized by shock [38]. Indeed, in the case of shock, the increase in blood potassium level is correlated with the importance of tissue hypoxia [39]. Hyperkalemia is secondary to the blockage of the Na-K ATPase pump [40] and the activation of the K_ATP_ channels triggered by cellular hypoxia [41, 42]. This activation leads to a hyperpolarization of the cellular plasmatic membrane and blocks voltage-dependent calcium channels. Hyperpolarization leads to a decrease in myocardial contractility and vasoplegia, reinforcing shock [43, 44]. This vicious circle, leading to death, was reproduced in this experimentation: uncompensated shock and hyperkalemia were observed in group TH. For these reasons, the rise in blood potassium observed in our study reflects the severity of tissue damages, and it can be reasonably hypothesized that it was associated with higher myosin and TF serum concentrations in group TH [45].

##### The role of myosin

A recent study demonstrated that myosin can bind factors Xa and Va, consistent with their ability to create a stable ternary complex called prothrombinase that promotes prothrombin activation [46]. Thus, a rise in myosin in group TH could promote thrombin generation, explaining normal ETPs despite the consumption of coagulation factors and fibrinolysis.

##### The role of TF

As previously described, the prothrombinase complex is composed of factor Va and factor Xa, and thrombin generation is directly dependent on its activity. As a consequence, a decrease in factor X should lead to a lowering in ETP. However, this lowering is limited in the presence of high levels of TF that promote activation of factor X [47]. These patterns were retrieved in groups C and TH. Indeed, a lowering in FX activity and ETP was observed in group C, reflecting a subtle impairment in the coagulation process due to cervical incisions, tracheostomy, and catheter insertions. In contrast, a lowering of FX without a decrease in ETP was observed in group TH. In this last group, a higher level of TF due to trauma should have limited the decrease in ETP.

##### Clinical implications

The observation of paradoxically normal ETPs in the TH group indicates the presence of procoagulant processes in case of trauma-related shock and is consistent with a recent study on humans [45]. The main advantage of this procoagulant mechanism in terms of survival could be to counterbalance the effect of coagulation factors depletion and aPC-mediated fibrinolysis. However, thrombosis is the price to pay to lower mortality [24]: a recent publication concluded that a procoagulant phenotype was a predictor of symptomatic venous thromboembolism after trauma [48]. These observations are consistent with the fact that, after several days, patients with ATC present higher mortality rates despite the restoration of normal blood pressure. Indeed, the presence of ATC is associated with multiple organ failures related to microcirculation defects [49–55].

### Fibrinogen plays a key role in ATC

In our study, fibrinogen concentration was dramatically decreased in the TH group at the end of the experimentation. At the same time, the mean ETP was higher in group TH than in group C. These results could be explained by the structure of fibrinogen, thrombomodulin, and thrombin. Indeed, fibrinogen and thrombomodulin have the same binding site on thrombin, the FRS site, suggesting a competitive inhibition of fibrinogen on the thrombin/thrombomodulin complex [56]. We, therefore, hypothesize that, in the case of ATC, a decrease in fibrinogen concentration could decrease thrombin consumption. At the same time and despite the preservation of normal thrombin concentrations, the lowering in fibrinogen concentration could decrease its competitive inhibition on the thrombin/thrombomodulin complex and activate the protein C pathway, reinforcing fibrinolysis. Also, we observed an inversed correlation between fibrinogen levels and aPTT, suggesting a protective role of fibrinogen against ATC. This result strengthens the hypothesis of a central role of fibrinogen in ATC’s pathophysiology [57], which could be explained by a decrease in the competitive inhibition of fibrinogen on the thrombin/thrombomodulin complex, and is coherent with the hypothesis of an increase in aPC leading to fibrinolysis.

### ATC can occur without platelet function impairment

Another hypothesis to explain the pathophysiology of ATC would be a loss of platelet function leading to a hemorrhagic phenotype on the early phase of trauma. According to this hypothesis, the burst in thrombin would cause diffuse platelet activation via their PAR receptors. This excessive activation would be followed by a refractory period characterized by a loss in platelet function. In this study, we observed no increase in thrombin generation and platelet response was similar in groups C, T, H, and TH at 90 min. These results invalidate the hypothesis of a decrease in platelet function mediated by thrombin as a key driver in ATC’s genesis. Similar results were observed in traumatized patients [22].

### Limitations

This experimentation was conducted on rats, and conclusions cannot be directly transposed on humans. Previous studies enlightened quantitative differences between the two species. In particular, clot formation is more efficient on rats [58]. However, hemostasis mechanisms, playing a crucial role in survival, are highly conserved. Indeed, key components such as cells, coagulation factors, and regulation mechanisms are similar in rats and humans [15, 57, 59–64]. It is therefore reasonable to assume that hemostasis disorders are also very close [1, 61, 65, 66]. Moreover, in this experimentation, low volumes of blood were sampled in order to avoid mimicking excessive bleedings, which would have biased the experiment. The small volumes of blood samples collected were insufficient to confirm all hypothesis developed in this experimentation, especially concerning fibrinolysis. All these hypothesis needs to be validated in clinical studies.

## Conclusion

**Fig. 4:**
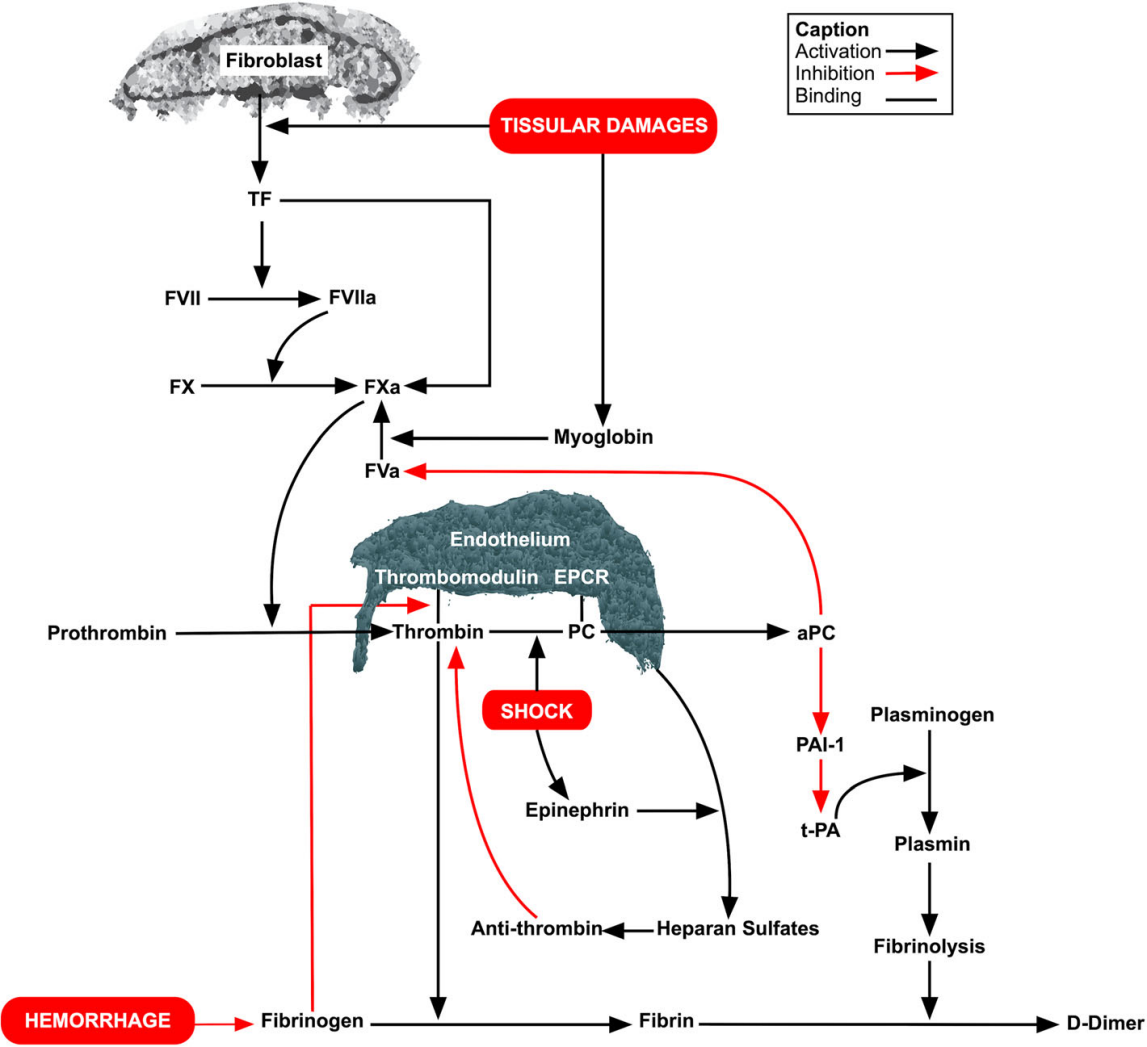
New considerations on pathways involved in acute traumatic coagulopathy. Hemorrhage leads to fibrinogen depletion and decreases its competitive inhibition on the thrombin/thrombomodulin complex, enhancing protein C activation. In addition, shock induces a decrease in thrombin clearance, also increasing thrombin/thrombomodulin interactions and protein C activation. The result is a hyperfibrinolysis triggered by aPC. Shock also lead to an increase in endogenous epinephrine, leading to heparan sulfate exposition on endothelial cells, activating antithrombin. The repression on coagulation mediated by antithrombin and activated protein C is counteracted by increases in tissue factor and myoglobin triggered by tissular damages, explaining the preservation of thrombin generation despite the expression of a hemorrhagic phenotype due to hyperfibrinolysis.

ATC resulted in a specific clinical-biological profile combining a hemorrhagic phenotype, the depletion of coagulation factors, and the preservation of thrombin generation. These results are consistent with excessive fibrinolysis mediated by aPC. The crucial role of fibrinogen in ATC was confirmed in this experimentation and could be explained by a decrease of its competitive inhibition on the thrombin/thrombomodulin complex, reinforcing fibrinolysis. The paradoxically preserved thrombin generation in this setting suggests a protective mechanism mediated by myoglobin and TF. We also observed that ATC could occur without significant impairment in platelet function. As a consequence, this experimentation led to a better understanding of ATC’s pathophysiology, which appears to be partially counterbalanced by survival-related mechanisms at the cost of an increase in thrombotic events. We propose a new conception concerning ATC’s pathophysiology based on these results (Fig. 4).

## Data Availability

The dataset used and analyzed during the current study is available from the corresponding author on reasonable request.

## Abbreviations

aPC: Activated protein C
aPTT: Activated partial thromboplastin time
ATC: Acute traumatic coagulopathy
DIC: Disseminated intravascular coagulation
ETP: Endogenous thrombin potential
MAP: Mean arterial pressure
PC: Protein C
PT: Prothrombin time
t-PA: Tissue plasminogen activator
TF: Tissue factor
TGT: Thrombin generation test
